# Beyond the Norm: A Case of Multiorgan Injury Triggered by Ibuprofen

**DOI:** 10.7759/cureus.46461

**Published:** 2023-10-04

**Authors:** Shawn Medford, Abdelwahab Jalal Eldin, Ahmed Brgdar, Lilian Obwolo, Ademola S Ojo, Constance Mere, Ahmed Ali

**Affiliations:** 1 College of Medicine, Howard University College of Medicine, Washington, DC, USA; 2 Internal Medicine, Howard University Hospital, Washington, DC, USA; 3 Nephrology, Howard University Hospital, Washington, DC, USA; 4 Oncology, Howard University Hospital, Washington, DC, USA

**Keywords:** covid 19, drug induced immune hemolytic anemia, drug-induced immune thrombocytopenia (ditp), ibuprofen induced injury, nsaid reactions

## Abstract

We report the case of a 71-year-old African American male with a history of chronic obstructive pulmonary disease (COPD), heart failure, vitiligo, penicillin allergy, and cocaine use, who presented with respiratory symptoms and was diagnosed with sepsis, COVID-19 pneumonia, exacerbation of COPD, and acute kidney injury (AKI). Treatment included antibiotics and high-dose steroids. The patient developed thrombocytopenia, autoimmune hemolytic anemia, acute liver failure, and interstitial nephritis associated with prolonged ibuprofen use. High-dose steroids and ibuprofen discontinuation led to significant improvement. This case highlights the rare occurrence of multiorgan injury from ibuprofen use, possibly aggravated by COVID-19, emphasizing the need for cautious non-steroidal anti-inflammatory drug (NSAID) use and close patient monitoring.

## Introduction

Non-steroidal anti-inflammatory drugs (NSAIDs) are generally safe and commonly used over-the-counter drugs. Rarely, they can lead to unpleasant severe adverse events. Reported events in the literature involve the gastrointestinal, hematological, hepatic, renal, cardiovascular, and integumentary systems. Multiorgan injuries are very rare [1]. In this report, we describe a case of severe multiorgan injury associated with the use of ibuprofen in the context of a COVID-19 infection.

## Case presentation

A 71-year-old African American male with a history of chronic obstructive pulmonary disease (COPD), heart failure with preserved ejection fraction, vitiligo, penicillin allergy (hives), and cocaine use presented to the emergency room (ER) with complaints of shortness of breath, cough, and pleuritic chest pain for three days. Vital signs indicated tachycardia (heart rate 103 beats per minute [bpm]), mild fever (temperature 100.4 °F), and oxygen saturation of 95% on 2 L oxygen. Lab findings showed a positive SARS-COV-2 polymerase chain reaction (PCR), leukocytosis, acute kidney injury (AKI), elevated liver enzymes, eosinophilia, and new thrombocytopenia. Chest X-ray revealed no infiltrates. The patient was admitted and initially managed for sepsis, COVID-19 pneumonia, COPD exacerbation, and AKI.

The patient was initially started on doxycycline and aztreonam for sepsis while awaiting blood culture results. High-dose steroids with 6 mg of dexamethasone were administered for COVID-19 pneumonia and continued for five days. The patient did not receive remdesivir due to elevated liver enzymes. Hematology consultation was sought for worsening thrombocytopenia (platelets of 21,000), and mild normocytic anemia with no bleeding. Anemia workup showed mildly elevated lactate dehydrogenase (LDH), normal serum haptoglobin, iron, transferrin, vitamin B12, and RBC folate levels. The coagulation panel showed mildly elevated partial thromboplastin time (PTT), prothrombin time (PT), and D-dimer for age. VQ scan had a low probability of pulmonary embolism (PE). Hepatitis C and HIV serologies were negative. Antinuclear antibody (ANA) was negative, and thyroid stimulating hormone (TSH) was within the normal range (Table 1). Peripheral blood smear showed normocytic anemia, no schistocytes, spherocytes, or rouleaux. There was marked thrombocytopenia with no clumping, leukocytosis, and an increase in eosinophils with no other abnormalities. 

**Table 1:** Initial laboratory investigations conducted upon first admission. MCV, mean corpuscular volume; LDH, lactate dehydrogenase; BUN, blood urea nitrogen; ALP, alkaline phosphatase; ALT, alanine transaminase; AST, aspartate transaminase; INR, international normalized ratio; PTT, partial thromboplastin time

Investigation	Result	Reference
Hemoglobin	12.8 g/dL	14.6-17.8 g/dL
MCV	97.9 fL	77.4-98 fL
Reticulocytes	3.34%	0.5%-2%
Haptoglobin	131 mg/dL	36-195 mg/dL
LDH	447 U/L	100-250 U/L
WBC	24.1	3.2 x 10E9 to 10.6 x 10E9
Eosinophil	22.5%	0-6.1%
Platelet count	74	177 x 10E9 to 406 x 10E9
Serum BUN	51	7-25 mg/dL
Serum creatinine	3.5 mg/dL	0.6-1.2 mg/dL
Serum bicarbonate	16 mEq/L	22-32 mEq/L
Troponin	0.15 ng/mL	0-0.03 ng/mL
ALP	300 IU/L	30-130 IU/L
ALT	>5,000 IU/L	0-55 IU/L
AST	8,711 IU/L	0-50 IU/L
Total bilirubin	6.8 mg/dL	0.2-1.2 mg/dL
Direct bilirubin	1.4 mg/dL	0-0.2 mg/dL
INR	2.6	1.12-1.46
Albumin	3.6 g/dL	3.2-5.5 g/dL
PTT	76 seconds	25-35 seconds
Triglycerides	184 mg/dL	<150 mg/dL

Eosinophilia workup was negative for stool ova, parasites, and Strongyloides. The patient had positive antineutrophil cytoplasmic antibodies (ANCA) but negative proteinase 3 (PR3) and myeloperoxidase (MPO). The blood culture had no growth, and antibiotics were then discontinued. He continued to improve and was weaned off oxygen. AKI and thrombocytopenia resolved, and he was discharged on day 7.

**Figure 1:**
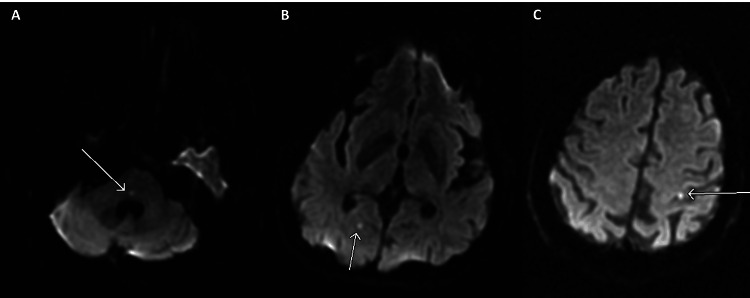
Brain MRI axial DWI: multiple acute punctuate infarcts: (A) right pons, (B) right occipital, and (C) left parietal. DWI, diffusion-weighted imaging; MRI, magnetic resonance imaging

**Figure 2:**
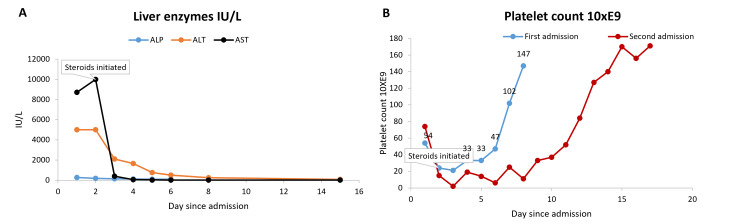
(A) Liver enzymes trend and (B) platelet count trend.

Four days later, the patient presented with slurred speech and was re-admitted due to acute multiple punctuate infarcts seen on the MRI brain (Figure 1). The patient was managed conservatively, and aspirin was withheld due to bleeding risk. He was admitted to the medical ICU for acute liver failure, marked by alanine transaminase (ALT) and aspartate transaminase (AST) exceeding 5,000 (Figure 2A), recurrent hypoglycemia, a recurrent episode of severe AKI with anuria, fractional excretion of sodium (FENa) of 0.2%, and nephrotic-range proteinuria (urine protein/creatinine ratio of 4.2 mg/g) necessitating intermittent hemodialysis. Additionally, the patient experienced a non-ST-segment elevation myocardial infarction (NSTEMI), persistent eosinophilia, normocytic anemia, and severe thrombocytopenia (Figure 2B). Further investigation revealed a positive direct antiglobulin test (DAT) for immunoglobulin G (IgG), but a negative result for C3. Furthermore, the urine drug screen was positive for cocaine.

**Figure 3:**
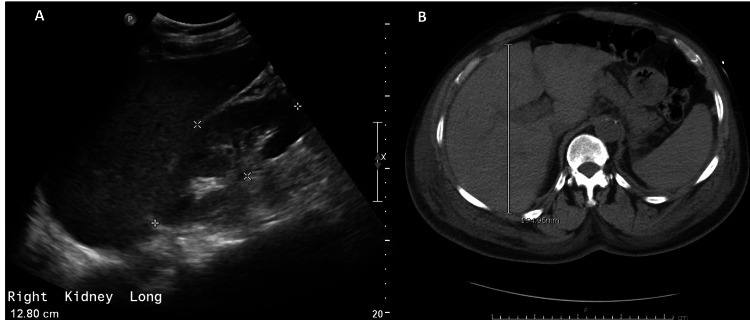
Hepatic assessment: (A) mild hepatic steatosis (abdominal ultrasound); (B) hepatomegaly (liver AP diameter: 19.4 cm) (abdomen CT scan). AP, anteroposterior; CT, computed tomography

Gastroenterology, infectious disease, and hematology teams evaluated the patient for acute liver failure. Laboratory tests indicated a low blood ethanol level, normal salicylates, and ceruloplasmin levels. Additionally, hepatitis A-E serology, Varicella Zoster virus (VZV), cytomegalovirus (CMV), rapid plasma reagin (RPR), West Nile virus (WNV) serology, anti-smooth muscle antibody, and anti-LKM (liver kidney microsomal) antibodies, all returned negative results. An abdominal ultrasound revealed hepatic and pancreatic steatosis (Figure 3A).

**Figure 4:**
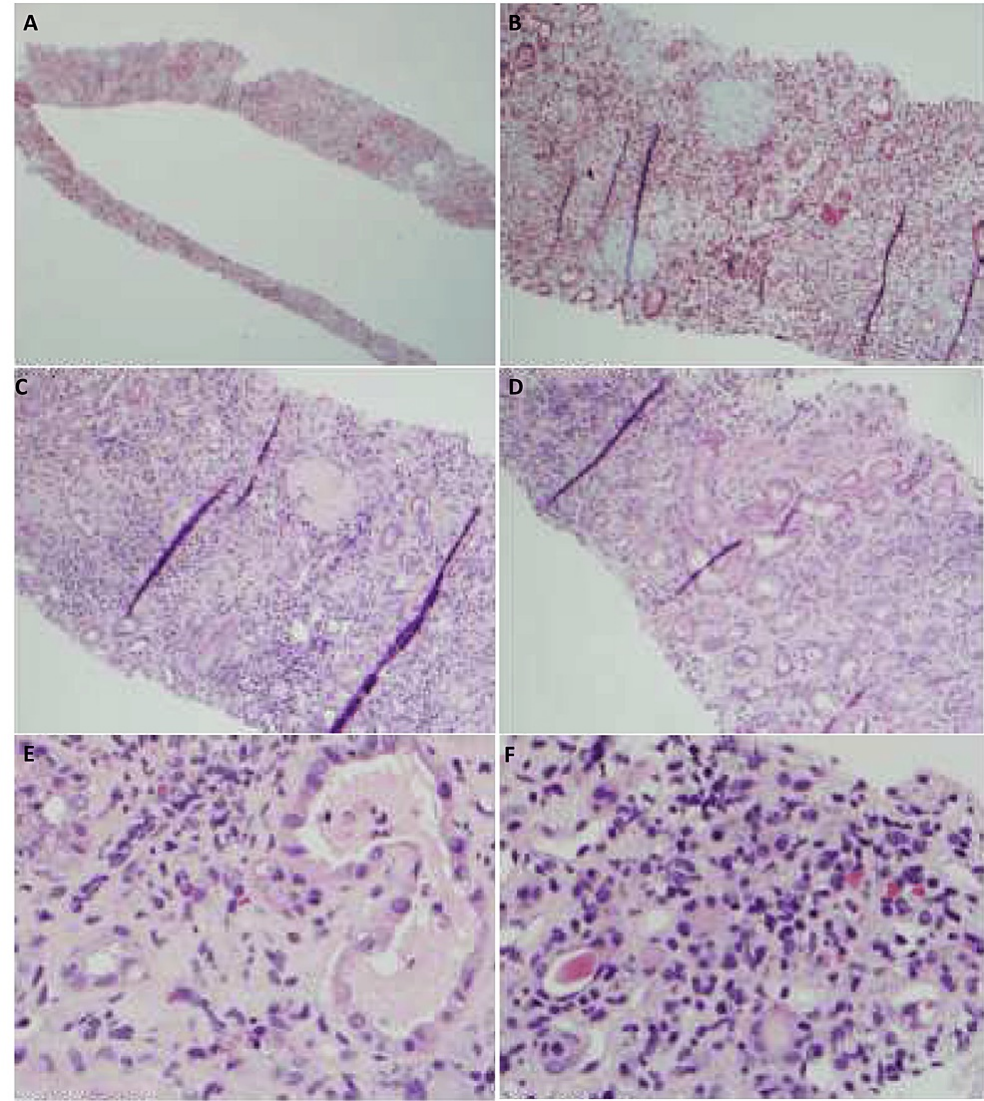
Renal biopsy. (A) Entire biopsy core showing interstitial fibrosis and tubular atrophy (IF/TA), Trichome. (B) Area of IF/TA with chronic inflammation, Trichome. (C and D) Patchy chronic inflammation in two different areas of evolving fibrosis, IF/TA, H&E. (E and F) Scattered eosinophils amidst lymphocytic infiltrate (in both images) and focal tubular injury (seen in E). H&E, hematoxylin and eosin

The patient developed recurrent thrombocytopenia, with a nadir of 2,000 platelets, without any apparent bleeding. The patient did not receive heparin, and he had a plasmic score of 4 (intermediate risk). A CT scan of the abdomen was suggestive of mild hepatomegaly and enteritis (Figure 3B). Bone marrow biopsy showed markedly hypercellular marrow, mature myeloid, and erythroid hyperplasia. Renal biopsy findings included resolving interstitial nephritis and focal tubular injury, moderate interstitial fibrosis and tubular atrophy, and mild arterionephrosclerosis changes with four out of 12 globally sclerosed glomeruli (Figure 4). The patient required 5 units of platelet transfusion. He was started on IV methylprednisolone (500 mg, tapered over two weeks), and IVIG (1 g/kg daily for three doses) due to suspicion of drug-induced immune hemolytic anemia (IHA) and thrombocytopenia given the reported history of over-the-counter high-dose ibuprofen use for weeks for abdominal pain, a positive combs test, vitiligo, eosinophilia, elevated IgE, and the recent pleasant response to steroids in the previous admission. He was started on IV methylprednisolone (500 mg, tapered over two weeks), and IVIG (1 g/kg daily for three doses) due to suspicion of drug-induced immune hemolytic anemia (IHA) and thrombocytopenia given the reported history of over-the-counter high-dose ibuprofen use for weeks for abdominal pain, a positive combs test, vitiligo, eosinophilia, elevated IgE, and the recent pleasant response to steroids in the previous admission. Following IV steroid administration, the patient had marked improvement in AST and ALT levels within a day, as well as platelet count, which continued to trend toward normal within several days (Figure 2). The patient remained hemodynamically stable throughout hospitalization and was advised to avoid ibuprofen indefinitely.

## Discussion

NSAID-induced organ injury is a relatively uncommon phenomenon, and its underlying mechanisms are attributed to various factors. This report presents an extraordinary case of acute liver failure accompanied by drug-induced immune thrombocytopenia (DITP), autoimmune hemolytic anemia, and interstitial nephritis following several weeks of over-the-counter ibuprofen use, coinciding with a concurrent COVID-19 infection. The patient exhibited a remarkable response to steroids and withdrawal of ibuprofen and achieved hematological recovery within days.

The estimated incidence of NSAID-induced liver injury ranges from approximately 1 to 10 cases per 100,000 prescriptions. The majority of cases are considered idiosyncratic reactions. Although ibuprofen-induced liver injury typically presents with mild ALT elevation, rare instances of acute liver failure and hypersensitivity reactions have been documented, occurring within days or weeks of drug use. Acute hepatitis, predominantly in a hepatocellular pattern, typically develops within one to three months of initiating therapy [2-4]. Our patient developed acute hepatitis after weeks of ibuprofen use, prompting an extensive workup to rule out other causes of thrombocytopenia. Significant improvement in liver enzyme levels was observed following the administration of intravenous (IV) methylprednisolone.

DITP and drug-induced immune hemolytic anemia (DIIHA) related to NSAIDs use are infrequent occurrences [5,6]. NSAID-induced DITP involves the production of drug-dependent antibodies that selectively bind to platelet membranes only in the presence of soluble drugs [7]. Similar to previously reported cases of ibuprofen-induced immune thrombocytopenia (ITP) and IHA, our patient achieved gradual hematological recovery with IV steroids, IVIG, and withdrawal of ibuprofen use [5,8,9].

NSAIDs can contribute to AKI through various pathophysiological processes, with acute interstitial nephritis being one of the most common manifestations, as observed in our patient [10]. Our patient developed severe AKI with anuria and severe hyperkalemia, necessitating emergent hemodialysis. His renal function gradually improved after discontinuing ibuprofen and facilitation of renal recovery with renal replacement therapy.

The potential impact of concurrent COVID-19 infection on NSAID-induced multiorgan injury warrants further investigation. Clinical reports have described COVID-19-induced interstitial nephritis, COVID-19-induced DITP, and COVID-19-induced AIHA [11-13]. However, no randomized controlled trials have been conducted to establish definitive relationships. Additional research is necessary to better understand the connection between COVID-19- and ibuprofen-induced organ injury.

## Conclusions

In conclusion, this case report presents a rare occurrence of multiorgan injury associated with ibuprofen use, accompanied by a concurrent COVID-19 infection. The patient showed a positive response to steroids and discontinuation of ibuprofen, resulting in complete recovery. Further research is needed to understand the potential influence of COVID-19 on ibuprofen-induced organ injury. Awareness of the potential adverse effects of NSAID use and careful monitoring are crucial.
